# A quasi-placebo may have a role in some randomised controlled trials

**DOI:** 10.1186/s13063-018-2496-8

**Published:** 2018-02-06

**Authors:** William P. Whitehouse

**Affiliations:** 1 School of Medicine, University of Nottingham, Nottingham, UK; 2 Paediatric Neurology, Nottingham Children’s Hospital, Nottingham University Hospitals NHS Trust, Nottingham, NG7 2UH UK

Dear Editor,

Migraine-prevention trials in children and young people have been disappointing. As chief investigator for P3MC, the placebo-controlled migraine-prevention trial of pizotifen and propranolol in the UK [1] that was abandoned because of poor recruitment, I have had a great deal to reflect on. And while having a placebo was not the main factor in the trial’s failure, I think that it could have contributed to some of the set-up sites recruiting no participants.

More recently, CHAMP, the placebo-controlled migraine-prevention trial of topiramate and amitriptyline in the USA [2] was also closed early, not because of poor recruitment, but because the high placebo responder rate made the study futile.

Great, a relatively cheap and safe intervention, the placebo, significantly reduced the headache frequency in 60% of participants. However, that outcome cannot be replicated clinically. The placebo is a trial intervention, which cannot exactly be replicated in clinical practice.

These abandoned trials failed to answer the important clinical questions about the efficacy of the two most prescribed treatments for preventing migraine in children in the UK and the USA respectively: ‘What is an effective treatment to recommend for this indication in these patients?’ This is particularly frustrating as they cost significant money, time and effort, and patient and parent/guardian investment.

Now there are a number of popular unproven treatments that are relatively cheap and accessible, for which there is no good-quality evidence and for which there probably never will be. Trials are expensive and the investment rightly requires a good chance that the results will be clinically useful, and if sponsored by pharma, a good chance of marketing success. These include low-dose aspirin [3], various drugs [4], and nutritional supplements and minerals [5], including riboflavin [6], and magnesium [7]. A variety of popular vitamin, mineral, and herbal supplement migraine treatments were reviewed by the National Institute for Health Care Excellence (NICE), who found riboflavin to be the most promising [8].

Randomised controlled trials (RCTs) use either an established comparator drug of proven efficacy or a placebo versus the investigational medicinal product (IMP). Both have drawbacks. The established drug may not have a sound evidence base itself, but more importantly, if the placebo is shown to have a high responder rate, as in the CHAMP study, clinicians are left with the message that the IMP is ineffective, or that although the placebo effect in the trial was actually impressive, it remains clinically inapplicable.

I propose a third way. Use a quasi-placebo as the placebo/comparator in the RCT of the target IMP. A quasi-placebo is a cheap, safe, pharmaceutical-grade medicine for which there is  insufficient data supporting efficacy, and is, for commercial reasons, unlikely to be tested in a definitive RCT. If the target IMP is superior to the quasi-placebo it is effective. If the target IMP is not, because the quasi-placebo had a high responder rate in the RCT, the drug used as the quasi-placebo could be used as a cheap and safe treatment in clinical practice. At least until a more effective alternative is found.

Using a quasi-placebo will be more acceptable to potential participants and their parents/guardians than a completely inert classical placebo. Furthermore, they know that if the quasi-placebo yields a high responder rate it can be used clinically whereas when a placebo yields a high responder rate, their participation will not lead to a clinically useful treatment.

The quasi-placebo should be used just like a classical placebo in the RCT: it should be indistinguishable from the target IMP, and participants, their parents/guardians, and clinical and trial staff should not know the treatment allocation. In addition, the trial protocol will require participants not to take over-the-counter preparations containing any of the IMP or quasi-placebo. This is a particular problem with medicines available over-the-counter. In practice this is a greater issue with acute rescue treatments rather than with regularly taken preventative treatments. These are important points, which have to be addressed in all clinical trials.

All treatments do exert placebo and nocebo effects. This is most easily seen in a placebo-controlled trial, where the placebo gives us an idea of the extent of the beneficial placebo effect exerted by the experimental treatment and the extent of the adverse or nocebo effect exerted by the experimental treatment. The problem faced by clinicians is how to use the placebo effect for the benefit of patients. Using a quasi-placebo enables the placebo effect to be exploited for patient benefit, as the quasi-placebo can be prescribed as a treatment, and is selected to be well tolerated, with a low risk of toxicity, low cost, and yet be pharmaceutically regulated.

With respect to future migraine-prevention trials in children, there are a number of candidate quasi-placebos, but perhaps riboflavin would be the best choice.

The problem of these migraine trials, low recruitment in P3MC, and the high placebo responder rate in CHAMP, were the inspiration for the concept. However quasi-placebos could be used effectively in clinical trials for other experimental treatments for other conditions. Their value, however, is principally limited to conditions where a high placebo responder rate is expected.

## Abbreviations

CHAMP: Placebo-controlled migraine-prevention trial of topiramate and amitriptyline
IMP: Investigational medicinal product
NICE: National Institute of Health and Care Excellence
P3MC: Placebo-controlled migraine-prevention trial of pizotifen and propranolol
RCT: Randomised controlled trial
UK: United Kingdom
USA: United States of America
